# The influence of individual and municipality characteristics on allocation of long-term care services: a register-based cross-sectional study

**DOI:** 10.1186/s12913-023-09641-y

**Published:** 2023-07-27

**Authors:** Lisa Victoria Burrell, Hanne Marie Rostad, Tore Wentzel-Larsen, Marianne Sundlisæter Skinner, Maren Kristine Raknes Sogstad

**Affiliations:** 1 Centre for Care Research, Norwegian University of Science and Technology (NTNU), Gjøvik, Norway; 2 Norwegian Centre for Violence and Traumatic Stress Studies (NKVTS), Gullhaugveien 1‑3, Oslo, 0484 Norway; 3 Centre for Child and Adolescent Mental Health, Eastern and Southern Norway, Gullhaugveien 1, Oslo, 0484 Norway

**Keywords:** Long-term care, Home care services, Nursing homes, Service allocation, Physical disability, Cognitive impairment, Register data, Multilevel analysis

## Abstract

**Background:**

Variation in service allocation between municipalities may arise as a result of prioritisation. Both individual and societal characteristics determine service allocation, but previous literature has often investigated these factors separately. The present study aims to map variation in allocation of long-term care services and investigate the extent to which service allocation is associated with characteristics related to the individual care recipient and the municipality.

**Methods:**

This cross-sectional study used register data from the Norwegian Registry for Primary Health Care on all 250 687 individuals receiving municipal health and care services in Norway on 31 December 2019. These individual level data were paired with municipal level data from the Municipality-State-Reporting register and information on the care models in Norwegian long-term care services, derived from a nationwide survey. Multilevel analyses were used to identify individual and municipal factors that were associated with allocation of home care, practical assistance and long-term stay in institutions.

**Results:**

In total, 164 634 people received home care services and 97 380 received practical assistance per 31 December 2019. Furthermore, 64 404 received both types of home-based services and 31 342 people had a long-term stay in an institution. Increased disability was strongly associated with being allocated more hours of home care and practical assistance, as well as allocation of a long-term institutional stay. The amount of home care and practical assistance declined with increasing age, but the odds of institutional stay increased with age. Care recipients living alone received more home-based services, and women had higher odds of a long-term institutional stay. Significant associations between the proportion of elderly in nursing homes and allocation of a long-term institutional stay and more practical assistance emerged. Other associations with municipalities’ structural characteristics and care service models were weak.

**Conclusions:**

The influence of individual characteristics outweighed the contribution of municipality characteristics, and the results point to a limited influence of municipality characteristics on allocation of long-term care services.

## Introduction

Long-term care services are under pressure: They face a range of challenges, and the care service landscape is changing [1]. National and international trends towards delivering health and care services closer to home have resulted in more patients being treated by the long-term care services as opposed to in hospitals. Consequently, the long-term care services are not only treating an increasing number of patients, but the patients also have more severe and complex illnesses [2]. Demographic changes are also increasing the burden on long-term care services through an increase in elderly people in need of care [3]. These challenges, in combination with limitations in personnel and financial resources, compel long-term care services to prioritise. Long-term care services are health and care services for people who are unable to care for themselves over a period of time and include help with everyday activities or help addressing medical needs. These services provide care throughout the lifespan, from health promotion and prevention to treatment, rehabilitation and palliative care. Long-term care is provided by several different professionals, at home, in assisted living facilities or in institutions [4].

Despite national guidelines, rules and regulations, the autonomy of Norwegian municipalities leaves room for flexibility in how they chose to meet local health and care challenges and organise and prioritise their long-term care services. As a consequence, both appropriate and unfavourable variation in long-term care services between municipalities will arise. Investigating factors that predict service allocation is a step towards understanding these differences. There is to date, however, limited knowledge of the factors impacting allocation of long-term care services. Førland and Folkestad (2016) proposed a range of factors that contribute to determine health and care service utilisation, including individual, relational, environmental and municipal factors. They postulate that factors related to the individual, such as health status, age, gender, opportunity to receive informal help and access to technological aids, together with municipal determinants related to resources combined determine home care service use [5]. Additionally, each municipality’s traditions, political ideology and strategic priorities are likely to influence service allocation. Previous research has, however, often investigated the influence of either individual [6–10] or municipal factors [11, 12] in isolation, and comprehensive investigation of several factors in combination is limited [5, 13, 14].

Previous studies have found that a patient’s disability level is a strong predictor of receiving care [6–8, 10, 13], and higher degrees of physical disability and cognitive impairment are associated with receiving more medical and practical help for patients in nursing homes [7] and home care services [6, 8, 13]. On the other hand, the specific diagnosis of the patient has reduced predictive value when disability is controlled for [6–8], implying that long-term care services may be mainly allocated based on the disability of the patient, and not the underlying diagnosis. When the effect of disability is taken into account, previous studies have not found age differences in the amount of help allocated to nursing home patients [7], or elderly people and intellectually disabled individuals receiving home care services [6]. There is, however, a tendency for younger people in general to receive more practical assistance compared to elderly patients [8]. Relational aspects of individuals’ lives also influence service allocation, and people living alone receive substantially more care than those living with a cohabitant [5, 6]. Receiving informal help from other sources may likewise influence service allocation [7, 13, 15].

When factors related to the individual patient are kept constant through studying municipalities’ allocation of services to a fictitious case, substantial variation between municipalities arise [12]. There is a tendency for municipalities with higher unrestricted revenues to use more resources per inhabitant and allocate more health and care services per inhabitant [5, 11–13]. Moreover, the availability of specialised healthcare services, such as nursing home units specialising in dementia care and rehabilitation and home care teams for dementia care and reablement, vary between municipalities [16]. Larger and more central municipalities have a higher availability of specialised care services than medium and small, less central municipalities. Such variations in resource use and service availability can probably be attributed to both favourable and unfavourable local adaptations. An example of favourable or necessary local adaptation of health and care services is more use of nursing homes in municipalities with very dispersed settlement, rendering home care highly resource-intensive [13]. Unfavourable variations occur if some municipalities have a prominent low use of resources or fail to provide mandated health and care services [11].

An important component of the explanatory framework by Førland and Folkestad to analyse service utilisation is the importance of factors both at the individual and municipal level [5]. Previous studies, however, seldom study all these factors combined, and knowledge of how these factors together impact the allocation of different health and care services is limited. Consequently, the present study aimed to map variation in allocation of long-term care services and investigate the extent to which service allocation is associated with characteristics related to the individual care recipient and the municipal context. We start by providing some information on the long-term care system in Norway.

### Organisation of long-term care services in Norwegian municipalities

In Norway, priorities and possibilities at the macro, meso and micro levels combined determine long-term care service provision and allocation. Municipalities are assigned the responsibility of long-term care services as a part of municipal health and care services, but the overall care policy is formulated by the government and resource allocation is largely nationally controlled [17, 18]. Long-term care in Norwegian municipalities is mostly financed through “unrestricted revenues” (tax revenues and government grants), which each municipality can administer freely, as well as fees and user payments. Municipalities are left to prioritise their health and care services within the national framework through planning and budgeting of resources. When individuals are in need of long-term care services, they file an application with the municipality, and this application can be accepted and result in a letter of service allocation where the type and scope of services are outlined [19]. The corner stones of long-term care are nursing homes, sheltered housing where people in need of care can live independently, and home-based services. These long-term care services are mandated by the government and are, hence, available in all Norwegian municipalities. As a result of the long-term care sector’s increased responsibility for providing health and care services to the population, additional and more specialised services have also emerged, such as nursing home units and home care teams specialising in dementia care, palliative care, psychiatric care and rehabilitation [16].

## Methods

The present register-based cross-sectional study used multilevel analyses to investigate the association of individual and municipal factors on the number of hours of home care services, the number of hours of practical assistance and the allocation of a long-term institutional stay.

The study utilised the IPLOS (Individual-based Nursing and Care Statistics) data from the Norwegian Register for Primary Health Care (NRPHC). This registration system has been mandatory in all Norwegian municipalities since 2006 and covers all care recipients of municipal health and care services, including recipients of nursing home and home-based services [20]. Through a cross-sectional design, we selected all individuals who received long-term care services on 31 December 2019. Secondly, information on gender, age and living arrangements were retrieved. Lastly, we added information on the disability level of the care recipients, as derived from a functional evaluation performed by healthcare personnel in 2019 and registered in NRPHC. The selection of participants was not restricted by age since the study aimed to investigate allocation of long-term care services for care recipients of all ages. Individuals registered with contradicting or clearly erroneous information, such as the date of service start after the date of the end of service, were excluded from the analysis. This selection resulted in 250 687 individuals who received one or more municipal health and care services on 31 December 2019.

The individual-level data outlined above were merged with information on the municipality where the individual received care. This municipal data was retrieved from the Municipality-State-Reporting database (KOSTRA), which includes information such as municipalities’ population size, income and expenses. All Norwegian municipalities report this data annually, and the information is publicly available on Statistics Norway’s website [20]. Lastly, information on care models in Norwegian long-term care services were merged with the above-mentioned individual- and municipal-level data. These long-term care models explore a way of describing how health and care services are provided in different municipalities. This data stems from a nationwide web-based survey performed by the researchers in 2019 where 277 municipalities (66% of Norwegian municipalities) answered a comprehensive questionnaire concerning the provision of long-term care services for adults. The different care service models were derived from a hierarchical cluster analysis which was based on municipalities’ level of specialised services, use of assistive technology in nursing homes and homecare, focus on disease prevention and health promotion, and focus on planning and coordination of care. The cluster analysis resulted in four municipality clusters or models. For further information about the survey study and models of care, see Rostad et al. (2023) [16].

### Dependent variables

The dependent variables investigated in the present study can be considered the pillars of long-term care services: home care services, practical assistance in the home and long-term stay in institutions.

The amount of home care was measured as the number of hours of home care allocated to an individual per week. These services involve a range of health and care services related to prevention, diagnostics, treatment and care for people who live at home, often through home nursing [21]. Likewise, the amount of practical assistance was also measured as the number of hours of practical assistance allocated to an individual per week. Practical assistance includes assistance at home to perform practical tasks such as cleaning, cooking, and shopping, as well as training in performing these tasks. User-controlled personal assistants are also included as a form of practical assistance [21]. In this article, we use home-based services as a collective term for home care and practical assistance. Long-term stay in an institution firstly refers to nursing homes, but may also include institutions for children and adolescents and institutions for people with substance use disorders [21]. This dependent variable was a dichotomous variable coded as yes/no.

### Independent variables

The individual level variables *age*, *gender* and *living arrangements* were included in the analyses. The variable living arrangements, whether individuals lived alone or not, was not included in the analyses of long-term stay in institutions given its lack of relevance when care recipients live in an institution and not at home. Furthermore, the disability level of the care recipient, as evaluated by healthcare personnel, was included in the analysis. This evaluation of disability is based on principles described by the World Health Organization’s classification of disabilities [22]. In total, the disability evaluation in NRPHC consists of 18 items that are evaluated on a scale from 1 to 5: 1 indicating no disability, 2 indicating some difficulty performing the task, but no need for assistance, and 3 or higher indicating increasing disability and need for assistance. A score of 9 – “not relevant” can also be given if, for example, a child is evaluated on aspects they are not expected to manage independently. Following the classification by the Norwegian Directorate of Health [23], the 18 items were categorised into five groups of highly related items: *Activities of Daily Living (ADL)* comprised personal hygiene, dressing, eating, using the toilet, and indoor and outdoor mobility. *Instrumental Activities of Daily Living (IADL)* included shopping, housekeeping and cooking. *Cognitive impairment* encompassed memory and communication, while S*ocial functioning* consisted of daily decision-making, social interaction, and behavioural control. Lastly, *Life management* consisted of maintaining one’s own health and attending to one’s own finances. This latter item related to managing finances was not included in the original classification by the Norwegian Directorate of Health but was classified by the researchers of this study. The items sight and hearing were excluded due to their lack of significance in the factor analysis that the above classification by the Norwegian Directorate of Health is based on [23]. Additionally, the disability level and need for assistance with regards to sight and hearing may be difficult to assess and score due to the use of effective aids such as glasses and hearing aids. The average score in each of the five groups was calculated.

The municipal level variables population size, centrality, unrestricted revenues, number of full-time equivalents (FTEs), the proportion of elderly in the municipality, and the proportion of elderly in nursing homes were included in the analyses. *Population size* was a continuous variable indicating the number of inhabitants in each municipality [24]. A municipality’s *centrality* was classified according to Statistics Norway’s index, which was based on the travel time to workplaces and important service functions, and was rated on a scale from 1) most central to 6) least central [25]. *Unrestricted revenues* was a continuous variable in Norwegian kroner (NOK) that was proportional to the population size of each municipality [26]. This variable indicated municipalities’ financial leeway as a measure of how much income the municipalities have at their disposal after covering fixed costs [27]. Moreover, the *number of FTEs* in the municipality’s health and care services was a continuous variable that was proportional to the population size of each municipality [28]. The *proportion of elderly in the municipality* was a continuous variable for the proportion of inhabitants in the municipality 80 years or older, measured per thousand (‰) [29]. Lastly, *the proportion of elderly in nursing homes* was a continuous variable for the percentage of inhabitants 80 years or older who reside in nursing homes [28]. To enhance understanding and interpretation of results, the continuous variables were modelled so that the regression coefficients and odds ratios from the multilevel analyses display the change in outcome following an increase per 1 000 inhabitants (population size), per 100 FTEs (number of FTEs), and per 10 000 NOK (unrestricted revenues). 10 000 NOK equals around 970 euros.

The *long-term care model* of the municipality, which was derived from the hierarchical cluster analysis of the survey data, consisted of four different care models. Municipalities classified as Care model 1 have a low to moderate focus on planning, coordination of care, disease prevention and health promotion, specialised services and assistive technology. This cluster consisted of 121 municipalities, where the majority are small municipalities. There were 105 municipalities classified in Care model 2, and these municipalities have a strong focus on planning and coordination of care, disease prevention and health promotion. Municipalities in Care model 3 have a strong focus on assistive technology, planning, and coordination of care, and 35 municipalities fitted within this cluster. Lastly, municipalities classified as Care model 4 have a large focus on planning, coordination of care, disease prevention and health promotion, high degree of specialised services and substantial use of assistive technology. This cluster consisted of 16, mainly large, municipalities. The long-term care model of the municipality was included as an indication of the historical traditions, political ideology and strategic priorities of the municipality.

### Statistical analyses

Firstly, we used descriptive statistics to describe the independent and dependent variables and their relationship through frequencies, crosstabs and measures of centrality and dispersion. Moreover, the unadjusted relationship between variables were investigated through Pearson’s correlations for continuous variables, phi coefficients for categorical variables, and point-biserial correlations for correlations between continuous and categorical variables [30].

Secondly, multilevel analyses, specifically mixed effects analyses, were used to investigate both individual and municipal factors that may influence allocation of long-term care services. Multilevel analyses can assess variation in allocation of services across and within municipal context, and hence identify and estimate the effects of individual and municipal characteristics on overall variability [31]. Clustering of outcome measures, i.e. that scores on outcome variables are not independent but where observations in the same cluster are more similar than observations in different clusters, is therefore accounted for in these analyses. As such, these models are generalizations of regression models for non-clustered data through estimation of random differences both between and within clusters [31]. Model 1 was an empty random intercept model without explanatory variables that was used to estimate the intraclass correlation coefficient (ICC). The ICC denotes the degree of clustering of services within municipalities and indicates the variation in service allocation that can be attributed to the municipal level [31]. Model 2 included individual characteristics, model 3 included municipality characteristics, and model 4 incorporated municipalities’ long-term care models. Possible improvements in predictive power from one model to the next were assessed using the likelihood ratio test where the improvement in deviance between the models is assessed together with the difference in degrees of freedom [31]. Results from statistical tests were deemed significant at a 5% significance level.

We conducted separate analyses for the number of hours of home care, the number of hours of practical assistance and allocation of long-term stay in an institution. Consequently, continuous dependent variables were assessed using multilevel linear analyses, specifically mixed effects models, and dichotomous dependent variables were assessed using multilevel logistic analyses, specifically generalised mixed effects logistic models. Note that the logistic analyses of allocation of long-term institutional stay used allocation of other municipal health and care services in NRPHC as reference group. The linear analyses of the amount of home care only included people who receive healthcare in the home, while the linear analyses of the amount of practical assistance only included people who receive practical assistance.

Construction of the dataset from individual data sources and statistical analyses were performed in R. Large datasets were handled by using the R package data.table, and multilevel models with random intercepts were estimated by using the glmmTMB package.

### Ethical approval

Register data from NRPHC was provided after the Regional Committee for Medical and Health Research Ethics in Norway gave an exemption to the requirement of confidentiality (reference number 76190). The survey study, which the long-term care model of the municipality is based on, was reported to the Norwegian Centre for Research Data (reference number 847216) before data collection. The Norwegian Centre for Research Data concluded that the processing of personal data in the project was in accordance with privacy legislation and they approved the process of gaining consent from participants. The informed consent form was attached to the email sent to potential respondents, where the email text stated that by completing the survey, the person consented to participation. Participation was confidential, and participants could only be identified by a few of the researchers. The survey study did not require approval from an ethical committee since it is not classified as medical and health research (defined as research on humans, human biological material and personal health information, which aims to generate new knowledge about health and diseases), and ethical approval was hence deemed unnecessary according to The Act on medical and health research (the Health Research Act)[32] in Norway. The study was performed in accordance with ethical standards laid down in the 1964 Declaration of Helsinki and its later amendments.

## Results

### Descriptive statistics

In total, 164 634 people received home care services and 97 380 received practical assistance per 31 December 2019. Of these care recipients, 64 404 received both types of home-based services. Furthermore, 31 342 people had a long-term stay in an institution at this time. Recipients of home care received on average 5.1 h (SD 14.2 h) of care every week, and recipients of practical assistance received on average 11.1 h (SD 30.5 h) per week. The median number of hours of home care and practical assistance of 1.5 and 1.0 indicate that most care recipients receive relatively few hours of help, while a minority receive extensive care.

Table 1 presents an overview of the sample characteristics of the care service recipients and their municipalities. There is a majority of female care recipients in both home-based services and long-term institutional stay, and the mean age of care recipients is substantially higher for residents in long-term institutions. Most recipients of home-based services live alone, especially recipients of practical assistance. The mean disability level of care recipients systematically varied between the different types of long-term care: recipients of home care had the lowest scores, recipients of practical assistance had higher scores, and the decidedly highest disability scores were found for recipients of long-term stay in institutions.

**Table 1:** Characteristics of care service recipients and their municipalities

N (%)^a^	Home care	Practical assistance	Long-term institutional stay
Gender			
Female	95 827 (58.23)	59 969 (61.60)	21 349 (68.14)
Male	68 732 (41.77)	37 390 (38.40)	9 981 (31.86)
Living arrangements			
Living alone	90 060 (61.34)	69 677 (77.10)	
Cohabiting	56 739 (38.65)	20 692 (22.90)	
Municipalities’ long-term care model			
Care model 1	26 179 (21.32)	15 069 (20.34)	4 357 (18.03)
Care model 2	58 818 (47.89)	36 128 (48.76)	12 055 (49.90)
Care model 3	18 397 (14.98)	10 178 (13.74)	3 264 (13.51)
Care model 4	19 421 (15.81)	12 723 (17.17)	4 484 (18.56)
**Mean (SD)** ^**b**^			
Age	66.12 (22.42)	68.37 (22.23)	83.89 (11.19)
ADL	1.86 (0.91)	2.04 (0.96)	3.49 (1.01)
IADL	2.60 (1.21)	2.98 (1.08)	4.55 (0.68)
Cognitive impairment	1.72 (0.86)	1.84 (0.95)	3.17 (1.03)
Social functioning	1.93 (0.91)	2.09 (1.04)	3.29 (0.99)
Life management	2.65 (1.08)	2.77 (1.26)	4.41 (0.80)
Centrality	3.16 (1.40)	3.06 (1.43)	2.95 (1.47)
Population size	93 687.17 (180 492.60)	111 824.03 (198 050.50)	135 240.70 (217 046.10)
Unrestricted revenues per inhabitant	59 128.52 (7 543.92)	59 235.75 (7 533.86)	59 672.38 (7 967.92)
Number of FTEs per 10 000 inhabitants	324.60 (92.35)	321.33 (96.37)	316.43 (96.97)
Proportion of elderly in the municipality (‰)	0.46 (0.12)	0.45 (0.12)	0.45 (0.12)
Proportion of elderly in nursing homes (%)	11.94 (3.31)	12.18 (3.30)	12.99 (3.01)

ADL – activities of daily living, IADL – instrumental activities of daily living, FTEs – full-time equivalents

^a^ N (%) for categorical variables

^b^ Mean and standard deviation (SD) for continuous variables

The mean population size was smallest for allocation of home care, larger for practical assistance and largest for long-term stay in institutions, which means that care recipients who receive home care more often live in smaller municipalities with fewer inhabitants whereas care-recipients living in institutions more often live in larger municipalities with more inhabitants. This may indicate that smaller municipalities more often allocate home-based services, while larger municipalities more frequently allocate long-term institutional stays. There were, however, small differences in the other structural characteristics and the long-term care model of the municipalities between the different types of long-term care.

### Unadjusted relationship – correlations

Analyses of correlations found several significant correlations between variables, and these can be found in the correlation matrix of Table 2. Notably, all individual characteristics were significantly correlated with long-term care services. A greater disability level of the care recipient was correlated with more hours of care and allocation of a long-term institutional stay, and the largest correlations were found between disability level and long-term institutional stay. The significant, albeit small, negative correlations between age and hours of care indicated that care recipients received fewer hours of home care and practical assistance the older they became. On the contrary, the significant positive correlation between age and long-term institutional stay showed that older individuals are more likely to be allocated a long-term institutional stay. Men were allocated more hours of home care and practical assistance, and women were more often allocated long-term institutional stays. Lastly, care recipients living alone received more hours of home-based services. In addition to the significant correlations between individual characteristics and long-term care, significant, but small correlations between municipality characteristics and long-term care were apparent.

There were strong positive correlations between the different types of disability, as well as significant correlations between age and disability. With age, the disability level of care recipients increased, expect for social functioning where the correlation with age was negative. Older care recipients were also more likely to live alone.

Strong correlations between the different municipality characteristics were also evident: more central municipalities had more inhabitants, less unrestricted revenues, fewer elderly inhabitants, and fewer employees in the long-term care services relative to the municipalities’ population size. Municipalities with higher unrestricted revenues had a higher proportion of elderly in nursing homes.

**Table 2:** Correlations of long-term care services with individual and municipality characteristics. Correlations significant at p < 0.001 are italicized

		1.	2.	3.	4.	5.	6.	7.	8.	9.	10.	11.	12.	13.	14.	15.	16.	17.
1.	Amount of home care	1																
2.	Amount of practical assistance	*0.085*	1															
3.	Long-term stay in an institution (0 = no/1 = yes)			1														
4.	ADL	*0.189*	*0.184*	*0.528* ^b^	1													
5.	IADL	*0.187*	*0.211*	*0.506* ^b^	*0.832*	1												
6.	Cognitive impairment	*0.143*	*0.208*	*0.485* ^b^	*0.638*	*0.670*	1											
7.	Social functioning	*0.162*	*0.277*	*0.427* ^b^	*0.584*	*0.630*	*0.796*	1										
8.	Life management	*0.170*	*0.234*	*0.493* ^b^	*0.647*	*0.729*	*0.755*	*0.779*	1									
9.	Age	*-0.009*	*-0.171*	*0.290* ^b^	*0.254*	*0.278*	*0.040*	*-0.184*	*0.026*	1								
10.	Gender (0 = female/1 = male)	*0.019* ^b^	*0.058* ^b^	*-0.074* ^a^	*-0.038* ^b^	-0.001 ^b^	*0.033* ^b^	*0.074* ^b^	*0.053* ^b^	*-0.191* ^b^	1							
11.	Living arrangements (0 = cohabitant/1 = alone)	*0.089* ^b^	*0.089* ^b^		*-0.137* ^b^	*-0.091* ^b^	*-0.135* ^b^	*-0.122* ^b^	*-0.098* ^b^	*0.170* ^b^	*-0.055* ^a^	1						
12.	Centrality (1: most central − 6: least central)	*0.038*	*-0.010*	*-0.032* ^b^	*-0.036*	*-0.041*	*-0.036*	*-0.041*	*-0.027*	*0.023*	0.006^b^	-0.007^b^	1					
13.	Population size	*-0.042*	*0.011*	*0.061* ^b^	*0.054*	*0.063*	*0.043*	*0.034*	*0.020*	*0.031*	*-0.009* ^b^	*0.017* ^b^	*-0.598*	1				
14.	Unrestricted revenues	0.002	*-0.007*	*0.028* ^b^	0.003	0.004	*-0.007*	*-0.030*	*-0.023*	*0.068*	-0.003 ^b^	0.002 ^b^	*0.399*	*0.191*	1			
15.	Number of FTEs	*0.033*	0.002	*-0.017* ^b^	*-0.025*	*-0.026*	*-0.026*	*-0.033*	*-0.020*	*0.036*	0.003 ^b^	0.005 ^b^	*0.716*	*-0.476*	*0.454*	1		
16.	Proportion of elderly in the municipality (‰)	*0.023*	*-0.013*	*-0.013* ^b^	*-0.023*	*-0.024*	*-0.032*	*-0.045*	*-0.028*	*0.068*	*-0.007* ^b^	*0.009* ^b^	*0.658*	*-0.466*	*0.425*	*0.761*	1	
17.	Proportion of elderly in nursing homes (%)	*-0.038*	-0.001	*0.099* ^b^	*0.039*	*0.044*	*0.022*	*0.011*	*0.017*	*0.058*	*-0.008* ^b^	0.002 ^b^	-0.003	*0.295*	*0.318*	*0.058*	*-0.014*	1

^a^ phi coefficient

^b^ point-biserial correlation

### Multilevel analyses

Tables 3, 4 and 5 present the results for the amount of home care, the amount of practical assistance and long-term stay in an institution, respectively. The first step in the analyses was an empty random intercept model without explanatory variables to identify the degree of clustering in service allocation within municipalities. The ICC for home care was 0.036, and the ICC for practical assistance was 0.031, indicating that 3.6% and 3.1% of the total variation in these services were at the municipal level. Moreover, 6.0% of the total variation in allocation of long-term stay in an institution were at the municipal level. Evidently, municipal clustering, i.e. the degree of resemblance in service allocation between care recipients belonging to the same municipality, is limited.

The second model in the chain of analyses included individual characteristics. The likelihood ratio tests of the differences in the deviances between the first and second models found that the inclusion of these individual characteristics significantly improved the models’ ability to predict allocation of all the investigated services (p < 0.001 for home care, p < 0.001 for practical assistance, and p < 0.001 for long-term institutional stay). For home care, a greater disability level of the care recipient was associated with allocation of more hours of help. Likewise, a greater disability level was significantly associated with allocation of a long-term institutional stay, as compared to other municipal health and care services. The disability level of the care recipient was positively associated with the amount of practical assistance, except for the measure of life management where greater disability was associated with less practical assistance. When comparing the different types of disability, ADL disability was the strongest predictor of the amount of home care, ADL disability and social functioning were the strongest predictors of practical assistance, and IADL disability and life management were the strongest predictors of long-term institutional stay. There were significant positive associations between living alone and the amount of home care and practical assistance; people living alone had a much greater chance of being allocated more help. Furthermore, there were significant negative associations between age and the amount of home care and practical assistance, and there was a significant positive association between age and long-term institutional stay. Care recipients had 8% higher odds of being allocated a long-term stay in an institution for every year they aged. For home care and practical assistance, there were no significant differences between male and female care recipients, while men had a significantly lower chance of being allocated a long-term institutional stay compared to other municipal health and care services.

The third model included municipal-level variables. The likelihood ratio tests found that the inclusion of municipality characteristics significantly improved the models’ ability to predict allocation of all the investigated services (p = 0.011 for home care, p < 0.001 for practical assistance, and p < 0.001 for long-term institutional stay). None of the municipal-level variables were significantly associated with the amount of home care. A significant positive association between the proportion of elderly in nursing homes in the municipality and the number of hours of practical assistance emerged. With regard to long-term institutional stay, there were significant negative associations with centrality and the number of FTEs, and significant positive associations with the proportion of elderly in the municipality and the proportion of elderly in nursing homes.

Finally, the fourth model included information about the long-term care model of the municipality, as an indication of the traditions, political ideology and strategic priorities of the municipality. The likelihood ratio tests between the third and fourth models found that including the long-term care model of the municipality did not significantly improve the predictive power of the models (p = 0.752 for home care, p = 0.102 for practical assistance, and p = 0.715 for long-term institutional stay). In general, the long-term care model of the municipality was not significantly associated with the allocation of long-term care services, with the exception that municipalities in Care model 4 allocated significantly more practical assistance compared to municipalities in Care model 1.

Only minor changes in the associations between service allocation and individual characteristics were evident when variables related to the municipality were added in models 3 and 4. When municipalities’ care models were included in model 4 for long-term institutional stay, the associations with centrality and the proportion of elderly in the municipality were no longer significant, while the associations with the number of FTEs and the proportion of elderly in nursing homes remained significant.

**Table 3:** Multilevel linear mixed effects models for the amount of home care

Fixed effect	Model 1 Empty model			Model 2 Individual characteristics			Model 3 Municipality characteristics			Model 4 Municipality care model	
	Estimate (95% CI)	p		Estimate (95% CI)	p		Estimate (95% CI)	p		Estimate (95% CI)	p
Intercept	4.83 (4.55;5.10)	< 0.001		-4.99 (-5.40;-4.58)	< 0.001		-5.58 (-7.12;-4.05)	< 0.001		-4.82 (-6.73;-2.90)	< 0.001
**Individual characteristics**											
ADL ^a^				4.33 (4.20;4.46)	< 0.001		4.33 (4.20;4.46)	< 0.001		4.14 (4.00;4.29)	< 0.001
IADL ^a^				0.20 (0.09;0.31)	< 0.001		0.20 (0.09;0.31)	< 0.001		0.24 (0.12;0.37)	< 0.001
Cognitive impairment ^a^				0.56 (0.44;0.69)	< 0.001		0.56 (0.44;0.69)	< 0.001		0.48 (0.33;0.62)	< 0.001
Social functioning ^a^				1.35 (1.21;1.48)	< 0.001		1.34 (1.21;1.48)	< 0.001		1.17 (1.02;1.32)	< 0.001
Life management ^a^				0.67 (0.57;0.78)	< 0.001		0.67 (0.56;0.77)	< 0.001		0.66 (0.54;0.78)	< 0.001
Age				-0.07 (-0.07;-0.06)	< 0.001		-0.07 (-0.07;-0.06)	< 0.001		-0.06 (-0.07;-0.06)	< 0.001
Gender (0 = female/1 = male)				-0.01 (-0.15;0.12)	0.847		-0.01 (-0.15;0.12)	0.855		-0.01 (-0.16;0.14)	0.894
Living arrangements (0 = cohabitant/1 = alone)				1.88 (1.73;2.02)	< 0.001		1.88 (1.74;2.02)	< 0.001		1.73 (1.57;1.89)	< 0.001
**Municipality characteristics**											
Centrality (1: most central − 6: least central)							0.32 (-0.02;0.67)	0.065		0.25 (-0.11;0.62)	0.177
Population size (per 1 000 inhabitants)							-0.004 (-0.010;0.003)	0.246		-0.003 (-0.01;0.00)	0.340
Unrestricted revenues (per 10 000 NOK)							-0.10 (-0.47;0.27)	0.607		-0.18 (-0.63;0.27)	0.435
Number of FTEs (per 100 FTEs)							0.29 (-0.03;0.62)	0.077		0.26 (-0.09;0.62)	0.147
Proportion of elderly in the municipality (‰)							-2.46 (-5.12;0.19)	0.069		-0.88 (-3.86;2.09)	0.561
Proportion of elderly in nursing homes (%)							0.01 (-0.06;0.07)	0.865		-0.02 (-0.09;0.06)	0.694
**Municipalities’ long-term care model**											
Care model 1										(ref.)	
Care model 2										-0.19 (-0.80;0.43)	0.548
Care model 3										0.24 (-0.62;1.09)	0.588
Care model 4										-0.27 (-1.43;0.90)	0.655
**Random effect**	**Variance component**			**Variance component**			**Variance component**			**Variance component**	
Municipal level variance	6.78 (2.60)			5.67 (2.38)			5.31 (2.31)			3.99 (2.00)	
Individual level variance	181.87 (13.49)			160.78 (12.68)			159.84 (12.64)			150.83 (12.28)	

^a^ Scale 1: very good − 5: very poor

**Table 4:** Multilevel linear mixed effects models for the amount of practical assistance

Fixed effect	Model 1 Empty model			Model 2 Individual characteristics			Model 3 Municipality characteristics			Model 4 Municipality care model	
	Estimate (95% CI)	p		Estimate (95% CI)	p		Estimate (95% CI)	p		Estimate (95% CI)	p
Intercept	10.30 (9.70;10.90)	< 0.001		6.59 (5.50;7.67)	< 0.001		1.86 (-1.78;5.51)	0.317		-0.52 (-5.65;4.61)	0.844
**Individual characteristics**											
ADL ^a^				5.90 (5.61;6.19)	< 0.001		5.91 (5.62;6.21)	< 0.001		6.15 (5.80;6.49)	< 0.001
IADL ^a^				1.97 (1.67;2.28)	< 0.001		1.97 (1.66;2.27)	< 0.001		2.01 (1.66;2.37)	< 0.001
Cognitive impairment ^a^				1.26 (0.95;1.56)	< 0.001		1.25 (0.95;1.56)	< 0.001		1.21 (0.85;1.57)	< 0.001
Social functioning ^a^				5.92 (5.58;6.26)	< 0.001		5.94 (5.60;6.27)	< 0.001		6.17 (5.78;6.57)	< 0.001
Life management ^a^				-0.39 (-0.64;-0.14)	0.002		-0.39 (-0.64;-0.14)	0.002		-0.34 (-0.63;-0.05)	0.021
Age				-0.41 (-0.42;-0.40)	< 0.001		-0.41 (-0.42;-0.40)	< 0.001		-0.41 (-0.42;-0.40)	< 0.001
Gender (0 = female/1 = male)				0.11 (-0.24;0.47)	0.537		0.10 (-0.26;0.45)	0.589		0.19 (-0.22;0.61)	0.358
Living arrangements (0 = cohabitant/1 = alone)				1.79 (1.38;2.19)	< 0.001		1.78 (1.38;2.19)	< 0.001		1.75 (1.28;2.22)	< 0.001
**Municipality characteristics**											
Centrality (1: most central − 6: least central)							0.11 (-0.69;0.90)	0.795		0.53 (-0.45;1.51)	0.290
Population size (per 1 000 inhabitants)							0.001 (-0.014;0.015)	0.923		-0.002 (-0.017;0.014)	0.820
Unrestricted revenues (per 10 000 NOK)							-0.22 (-1.09;0.65)	0.616		0.18 (-1.01;1.37)	0.765
Number of FTEs (per 100 FTEs)							0.55 (-0.20;1.32)	0.151		0.16 (-0.77;1.10)	0.731
Proportion of elderly in the municipality (‰)							0.39 (-5.79;6.58)	0.901		-2.38 (-10.31;5.54)	0.556
Proportion of elderly in nursing homes (%)							0.27 (0.11;0.42)	< 0.001		0.27 (0.07;0.47)	0.008
**Municipalities’ long-term care model**											
Care model 1										(ref.)	
Care model 2										0.59 (-1.06;2.24)	0.483
Care model 3										0.19 (-2.10;2.48)	0.870
Care model 4										3.95 (0.82;7.08)	0.013
**Random effect**	**Variance component**			**Variance component**			**Variance component**			**Variance component**	
Municipal level variance	28.27 (5.32)			29.72 (5.45)			27.75 (5.27)			28.59 (5.35)	
Individual level variance	882.98 (29.72)			617.55 (24.85)			617.97 (24.86)			644.71 (25.39)	

^a^ Scale 1: very good − 5: very poor

**Table 5:** Multilevel logistic generalised mixed effects models for long-term stay in an institution

Fixed effect	Model 1 Empty model			Model 2 Individual characteristics			Model 3 Municipality characteristics			Model 4 Municipality care model	
	OR (95% CI)	p		OR (95% CI)	p		OR (95% CI)	p		OR (95% CI)	p
**Individual characteristics**											
ADL ^a^				1.27 (1.24;1.30)	< 0.001		1.27 (1.24;1.30)	< 0.001		1.24 (1.21;1.28)	< 0.001
IADL ^a^				2.14 (2.07;2.22)	< 0.001		2.13 (2.06;2.21)	< 0.001		2.16 (2.07;2.25)	< 0.001
Cognitive impairment ^a^				1.11 (1.08;1.14)	< 0.001		1.11 (1.08;1.14)	< 0.001		1.09 (1.06;1.13)	< 0.001
Social functioning ^a^				1.46 (1.42;1.52)	< 0.001		1.46 (1.41;1.51)	< 0.001		1.48 (1.42;1.54)	< 0.001
Life management ^a^				2.09 (2.03;2.16)	< 0.001		2.10 (2.03;2.16)	< 0.001		2.10 (2.02;2.17)	< 0.001
Age				1.083 (1.081;1.084)	< 0.001		1.083 (1.081;1.084)	< 0.001		1.081 (1.079;1.083)	< 0.001
Gender (0 = female/1 = male)				0.83 (0.80;0.87)	< 0.001		0.83 (0.80;0.87)	< 0.001		0.85 (0.82;0.89)	< 0.001
**Municipality characteristics**											
Centrality (1: most central − 6: least central)							0.90 (0.82;0.98)	0.018		0.94 (0.84;1.05)	0.278
Population size (per 1 000 inhabitants)							1.00 (0.99;1.00)	0.902		1.00 (0.99;1.00)	0.982
Unrestricted revenues (per 10 000 NOK)							1.02 (0.93;1.13)	0.635		1.04 (0.91;1.18)	0.578
Number of FTEs (per 100 FTEs)							0.86 (0.79;0.94)	< 0.001		0.86 (0.78;0.96)	0.006
Proportion of elderly in the municipality (‰)							2.95 (1.46;5.95)	0.003		2.29 (0.93;5.63)	0.070
Proportion of elderly in nursing homes (%)							1.18 (1.16;1.20)	< 0.001		1.16 (1.13;1.18)	< 0.001
**Municipalities’ long-term care model**											
Care model 1										(ref.)	
Care model 2										1.06 (0.88;1.28)	0.527
Care model 3										0.96 (0.74;1.25)	0.784
Care model 4										1.17 (0.82;1.67)	0.378
**Random effect**	**Variance component**			**Variance component**			**Variance component**			**Variance component**	
Municipal level variance	0.21 (0.46)			0.90 (0.95)			0.37 (0.61)			0.37 (0.61)	

^a^ Scale 1: very good − 5: very poor

## Discussion

For Norwegian long-term care recipients, increased disability was strongly associated with being allocated more home care and practical assistance, as well as a long-term institutional stay. Moreover, the amount of home care and practical assistance declined with increasing age, but the chance of institutional stay increased with age. Care recipients living alone received more home-based services, and women had a higher chance of being allocated a long-term institutional stay. Significant associations between the proportion of elderly in nursing homes and allocation of a long-term institutional stay and more practical assistance emerged. Other associations with municipalities’ structural characteristics and care service models were weak. Evidently, individual conditions are most influential in allocation of long-term care services, while the influence of the municipalities’ structural characteristics and model of care is more limited.

### The importance of individual characteristics

The large influence of the disability level of the care recipients on service allocation is in line with previous national and international studies [6, 7, 10]. The disability level and concomitant need for care is considerably higher for people who have a long-term institutional stay compared to recipients of home-based services. As the disability of the care recipient increases, they are allocated more home-based services until their functioning has deteriorated to such an extent that they are allocated an institutional stay. Disability in ADL and social functioning are the disability types most strongly related to the amount of home-based services. A decline in activities performed several times during the day, such as dressing, personal hygiene, eating and using the toilet probably leads to a drastic increase in both the frequency and duration of visits from long-term care services. At a minimum, help with these basic daily activities needs to be provided, regardless of the financial resources and number of employees in the municipality. The strong association between social functioning, i.e. daily decision-making, social interaction, and behavioural control, and the amount of home-based services may be a result of the large group of younger care recipients with intellectual disabilities. This is especially apparent for practical assistance. Correspondingly, a previous Norwegian study found that care recipients with an intellectual disability receive more practical assistance than care recipients with other disorders, given the same disability level [8]. With respect to the different types of disability, the strongest predictors for allocation of long-term institutional stay were IADL (shopping, housekeeping and cooking) and life management (maintaining one’s own health and attending to one’s own finances). Individuals who are unable to perform these tasks will have a rapid decline in health and quality of life, and an apparent marginalisation in society. They quickly become unable to live alone without help, especially if coupled with extensive somatic or psychiatric health issues.

The physical disability of care recipients, as measured through ADL and IADL, is clearly important in determining service allocation. This is in line with previous research reporting that “an increase in physical disability increased the provision of public care to a greater extent than an increase in cognitive impairment” [6]. Alternatively, we can argue that ADL and IADL disability is a result of both the physical and cognitive functioning of the individual. Therefore, the importance of ADL and IADL to service allocation can be an indication of the importance of both physical and cognitive ability. Likewise, difficulty in life management can be a manifestation of both physical and cognitive disability. Confusion, lack of understanding, declining memory and poor communication can come to light through an inability to perform daily tasks and basic housekeeping. Healthcare personnel may find evaluations of memory and communication in care recipients challenging, and they can have more difficulty understanding the care needs of recipients with cognitive impairments as opposed to the care needs of recipients with somatic impairments [6]. In addition, declining cognitive abilities can be harder to identify than declining physical abilities and will hence more seldom prompt a need for a new evaluation of the care recipient’s disability level. Indeed, the NRPHC has been criticised for not being able to capture and register the true cognitive capacity of care recipients [33]. As a result, service allocation based on the disability evaluations that are reported to NRPHC may run the risk of underestimating the care need of the recipient.

In addition to the disability level of the care recipient, several other individual characteristics were strongly related to service allocation. The chance of a long-term institutional stay increased with age, while the amount of home-based services decreased with age. A positive association between age and institutionalisation is also found in other western countries [10]. Whereas older people in need of care are more often allocated a stay in nursing homes, younger care recipients with an extensive need for help are, in line with national policy, provided comprehensive home-based care [8, 13]. These younger care recipients often suffer from intellectual disability or psychiatric disorders and constitute a growing population in need of extensive and long-lasting care [34]. In accordance with previous studies [6, 13, 35], care recipients who live alone received more home care and practical assistance. Family and friends constitute a large source of help for individuals with a reduced functioning and increased need of care, with regard to both practical and health-related activities [36]. Lastly, women had a higher chance of receiving a long-term institutional stay compared to men. Previous studies from Norway and the U.S., however, did not report differences in nursing home admission between males and females [9, 10]. On the other hand, no gender differences were evident for home care and practical assistance in the present study, and this is in accordance with a previous study of Norwegian care recipients [8].

### The lesser influence of municipality characteristics

In general, the influence of individual characteristics substantially outweighed the contribution of municipality characteristics, and the results point to a limited influence of municipality characteristics on allocation of long-term care services. This is not the case in all Western countries [14], and may be a result of the Nordic welfare model. The Norwegian government’s strategic plans for the health and care sector highlight the importance of individual needs and preferences in the distribution and development of the long-term care services [37, 38]. The present results may imply that Norway is on route to reach its goal to allocate services mainly based on individual needs and preferences. This is also a step in the direction of universal access and fair distribution of services, which contributes to high-quality health and care services [39].

Since the present study only includes people who receive some form of health- and care services from the municipality, the study cannot conclude whether municipality characteristics influence the threshold to receive care at all. The variables that influence who are allocated care and who do not receive any services from the municipality are still unknown. Likewise, variables related to structural characteristics and the long-term care model of municipalities may influence allocation of other services or the quality of the allocated services. These unanswered questions are important avenues for future studies.

We argue that the limited influence of municipality characteristics is partly because the leeway of municipalities in allocating long-term care services has diminished, and the variability between municipalities lessened over time. Sandwiched between government regulations and limited resources, the flexibility of municipalities in allocating services has become constrained. According to Øydgard (2018), case workers who are responsible for allocating services are governed by the ruling principles of necessary, justifiable healthcare and the lowest effective care level. These principles are tied to overarching legal provisions and national principles for allocation of health and care services [19], rendering the individuals working in the allocation offices mere administrators of national policies. In addition, the limited associations between service allocation and municipality characteristics can be explained by the municipalities effectively trying to adapt to the allocation level of other municipalities. This type of benchmarking is made possible through the plethora of publicly available municipal information that is published every year, allowing municipalities to compare for example their income, spending, number and educational level of healthcare staff and service prioritisation [40]. Municipalities eventually find an adequate level of service allocation that is comparable to other similar municipalities, effectively reaching a shared national consensus.

The tendency for municipalities with higher proportions of elderly in nursing homes to more easily allocate long-term institutional stays may be due to these municipalities’ better finances given the correlation between the proportion of elderly in nursing homes and the unrestricted revenues of the municipality. Arguably, richer municipalities use their financial leeway to fund more beds in nursing homes as opposed to sheltered housing or home-based services. Caring for a patient in a nursing home with 24-hour staffing has been regarded as more expensive than sheltered housing where care services are provided as home-based services [41]. Municipalities with restricted means may hence choose to develop sheltered housing for care recipients with an extensive need for care, whereas municipalities with more generous resources often opt for traditional nursing homes. Since sheltered housing is thought to give care recipients more freedom, independence, privacy, dignity and flexibility, this solution is preferred by some municipalities, especially for younger care recipients. It is, however, questionable how suitable such housing is for frail elderly people and people suffering from dementia [41]. Although nursing homes may be a good option for many municipalities, extensive use of nursing homes may impede the development of robust home care, rendering the municipality less able to adapt to future needs and scarce resources.

The significant negative association between the number of FTEs and allocation of long-term institutional stays was somewhat surprising. The number of FTEs is, however, not a direct reflection of the number of health care personnel working in contact with patients, as many full-time equivalents are also employed in administrative and leader positions within the municipality. In addition, the number of FTEs in the municipalities’ health and care services is connected to all care services, and some municipalities may allocate more personnel and resources to services on a lower care level.

In the present study, municipalities in Care model 4 allocated more practical assistance than municipalities in Care model 1, and municipalities with a higher proportion of elderly in nursing homes allocated more practical assistance. Municipalities in Care model 4 and municipalities with a higher proportion of elderly in nursing homes are often large and central. The more extensive allocation of practical assistance in these large and central municipalities may be because they have more relocated inhabitants [42] with fewer family members in close proximity who can help them with everyday tasks and health-related activities. Indeed, a comprehensive European study reported that a shorter distance to a person’s social network increased the likelihood of receiving informal care [15]. A lack of informal care may not be completely covered by formal health and care services, and care recipients with a lacking social network are especially at risk of care poverty – “the deprivation of adequate coverage of care needs resulting from interplay between individual and societal factors” [43]. Given the present study’s results and the lack of nationally representative datasets in the study of unmet needs [43], future register-based studies should examine care poverty further. Another implication of the present study results is that future studies investigating variation between municipalities should include individual characteristics to gain meaningful insight.

### Strengths and limitations

To our knowledge, the present study is the first to investigate the influence of both individual and municipal characteristics on allocation of both home-based services and institutional care in Norway. The large number of individuals included in the study ensures high statistical power and a thorough investigation. Coupled with the national coverage of the registers utilised, the present study has high external validity. A limitation of the study results is that potential discrepancies between the allocation decisions and actual practice will not be reflected in the registers. In cases where healthcare workers digress from the official allocations, for example by providing more or other home-based services than what is stated in the official allocation [44], the study will not incorporate this deviance. Furthermore, even though IPLOS data has a relatively high data quality [45], the NRPHC is not safeguarded against possible errors, misclassifications or omissions.

## Conclusions

In conclusion, the study results identified the individual condition of the care recipient as most influential in predicting allocation of long-term care services. Characteristics related to the municipalities’ structural characteristics and care service model are of limited importance compared to individual factors. Consequently, allocation of long-term care services in Norway seems to be primarily based on individual needs and preferences.

## Data Availability

The data that support the findings of this study are available from the Norwegian Directorate of Health but restrictions apply to the availability of these data, which were used under license for the current study, and so are not publicly available. Data are however available from the authors upon reasonable request and with permission of the Norwegian Directorate of Health.

## Abbreviations

IPLOS: Individual-based Nursing and Care Statistics
NRPHC: Norwegian Registry for Primary Health Care
KOSTRA: Municipality-State-Reporting
ADL: Activities of Daily Living
IADL: Instrumental Activities of Daily Living
FTEs: Full-time equivalents
NOK: Norwegian kroner
ICC: Intraclass correlation coefficient
SD: Standard deviation
ORs: Odds ratios
95%: CIs 95% confidence intervals
